# Exercise Training Reverses Skeletal Muscle Atrophy in an Experimental Model of VCP Disease

**DOI:** 10.1371/journal.pone.0076187

**Published:** 2013-10-09

**Authors:** Angèle Nalbandian, Christopher Nguyen, Veeral Katheria, Katrina J. Llewellyn, Mallikarjun Badadani, Vincent Caiozzo, Virginia E. Kimonis

**Affiliations:** 1 Department of Pediatrics, Division of Genetics and Metabolism, University of California Irvine, Irvine, California, United States of America; 2 Department of Physiology and Biophysics, University of California Irvine, Irvine, California, United States of America; 3 Department of Orthopedics, University of California Irvine, Irvine, California, United States of America; Louisiana State University Health Sciences Center, United States of America

## Abstract

**Background:**

The therapeutic effects of exercise resistance and endurance training in the alleviation of muscle hypertrophy/atrophy should be considered in the management of patients with advanced neuromuscular diseases. Patients with progressive neuromuscular diseases often experience muscle weakness, which negatively impact independence and quality of life levels. Mutations in the *valosin containing protein* (*VCP*) gene lead to Inclusion body myopathy associated with Paget's disease of bone and frontotemporal dementia (IBMPFD) and more recently affect 2% of amyotrophic lateral sclerosis (ALS)-diagnosed cases.

**Methods/Principle Findings:**

The present investigation was undertaken to examine the effects of uphill and downhill exercise training on muscle histopathology and the autophagy cascade in an experimental VCP mouse model carrying the R155H mutation. Progressive uphill exercise in VCP^R155H/+^ mice revealed significant improvement in muscle strength and performance by grip strength and Rotarod analyses when compared to the sedentary mice. In contrast, mice exercised to run downhill did not show any significant improvement. Histologically, the uphill exercised VCP^R155H/+^ mice displayed an improvement in muscle atrophy, and decreased expression levels of ubiquitin, P62/*SQSTM1*, LC3I/II, and TDP-43 autophagy markers, suggesting an alleviation of disease-induced myopathy phenotypes. There was also an improvement in the Paget-like phenotype.

**Conclusions:**

Collectively, our data highlights that uphill exercise training in VCP^R155H/+^ mice did not have any detrimental value to the function of muscle, and may offer effective therapeutic options for patients with VCP-associated diseases.

## Introduction

Inclusion body myopathy associated with Paget's disease of bone and Frontotemporal dementia (IBMPFD) and 2% of familial amyotrophic lateral sclerosis (fALS) cases are caused by mutations in the *valosin containing protein* (*VCP*) [1], [2]. IBMPFD patients display rimmed vacuoles and TAR DNA-binding protein-43 (TDP-43)-positive ubiquitinated inclusion bodies in muscle [3]–[6]. The myopathy is characterized by muscle weakness and atrophy of skeletal, pelvic and shoulder girdle muscles. ALS patients demonstrate asymmetric focal weakness of the extremities or bulbar muscles which then spreads to the adjacent anatomical regions [7].

With greater than 27 mutations having been identified, VCP-associated disease is increasingly being recognized worldwide [8] in families from Germany [9], [10], France [11], Austria [12], Italy [13], [14], the UK [15], Australia [16], Brazil [17], Korea and the US [18], [19]. Most mutations occur in the ubiquitin binding domain of VCP with the R155H mutation being the most common mutation, accounting for approximately half of the affected individuals [2], [20], [21]. VCP is a member of the type II AAA family and possesses two ATPase domains [22]. VCP plays a critical role in a broad range of cellular activities including homotypic membrane assembly, endoplasmic reticulum-associated degradation of proteins (ERAD), the ubiquitin-proteasome system (UPS), cell cycle regulation, DNA repair, prevention of polyglutamine aggregation, autophagosome maturation in autophagy and more recently in mitophagy (for review [21]). Further insight into these cellular and molecular mechanisms may help in understanding and in investigating specific therapeutic targets for these patients.

The effects of exercise physiology have been investigated in numerous mouse models, most commonly in the *mdx* mouse system for Duchenne Muscular Dystrophy (DMD). Several reports have demonstrated that exercise training improves muscle function and ameliorates the disease state of the *mdx* animals [23]–[25]. Exercise physiology has also been reported to enhance muscle mitochondrial biogenesis and protection for mice with mitochondrial myopathies [26], [27]. Chronic aerobic exercise has been shown to stimulate the 5′-AMP-activated protein kinase (AMPK) activity [28]–[30]. Similarly, the beneficial effects of endurance training have been demonstrated in the transgenic nemaline mouse model of the extensor digitorum longus (EDL) muscle, revealing a novel myofiber repair mechanism [31]. Prevention of oxidative stress and overactivity in the ubiquitin-proteasome system in heart failure-induced myopathy in a mouse model was recently reported [32]. Endurance training also prevented TNF-α induced loss of force in the diaphragm of C57Bl6 mice [33]. In the present study, we evaluated the effects of exercise training and explored their potential therapeutic efficacy in our VCP disease model. We observed that treadmill endurance training improved muscle strength and mass, depicted a decreased number of inclusion bodies and apoptotic cells, decreased the levels of the autophagy cascade markers, and overall highlights the translational value of clinical intervention for patients with VCP disease.

## Materials and Methods

### Ethical statement

All experiments were done with the approval of the Institutional Animal Care and Use Committee (IACUC) of the University of California, Irvine (UCI) (IACUC Protocol #2007-2716-2), and in accordance with the guidelines established by the National Institutes of Health (NIH). Animals were housed in the vivarium and maintained under constant temperature (22°C) and humidity with a controlled 12∶12-hour light-dark cycle. Mice were provided standard rodent chow (Harlan Teklad Rodent Diet, Madison, WI) and water *ad libitum*. All of the mice were confined to their individual cages throughout the study.

### Treadmill Exercise Protocol

Generation, genotyping and characterization of the heterozygous VCP^R155H/+^ and WT animals has been described previously [34], [35]. Baseline measurement studies were performed on the VCP^R155H/+^ and WT mice prior to priming the mice for the exercise studies. The mice aged 18 months were divided into four groups of 8–10 mice each for uphill and downhill exercises: (a) VCP^R155H/+^ mice without exercise, (b) VCP^R155H/+^ mice with exercise, (c) WT littermates without exercise and (d) WT littermates with exercise. The mice in the exercise group were run on the Exer 3/6 treadmill (Columbus Instruments, Columbus, OH), consisting of six running lanes and an electrical stimulus system composed of shock grids. The following work load protocol was implemented for 6 weeks with exercise performed 3 days per week: *Week 0*: mice were primed at a speed of 5 M/min with a 0 degree inclination or declination for 10 min; *Week 1*: mice ran at a speed of 10 M/min with a 5 degree inclination or declination for 20 min; *Week 2*: mice ran at a speed of 12 M/min with a 10 degree inclination or declination for 30 min; *Weeks 3–6*: mice ran at a speed of 12 M/min with a 15 degree inclination or declination for 30 min. The control mice were placed in a different cage for 10–30 minutes as a sham stimulus according to the aforementioned schedule.

### Measurement Studies

Weights and survival points from the WT and VCP^R155H/+^ exercise (EX) and sedentary (SED) animals was measured on a weekly basis to follow body mass development. Mice were placed on the Rotarod apparatus before and after the exercise protocol, accelerating from 4 to 40 rpm in 5 minutes. Mice went through three trials with 45-minute to 60-minute inter-trial intervals on each of two consecutive days. The purpose of the first day was for mice to acclimate to the apparatus, and then during the second day, Rotarod performance was recorded (in seconds). Muscle strength of the forelimbs of mice was measured using a Grip Strength Meter apparatus (TSE Systems Gmbh, Hamburg, Germany). Briefly, mice were held from the tip of the tail above the grid and gently lowered down until the front paws grasped the grid. The animal was brought to an almost horizontal position and pulled back gently, but steadily until their grip was released. Hind limbs were kept free from contact with the grid. The maximal force achieved by the animal was recorded and each animal underwent 5 testings with 5 minute breaks in between each measurement.

### Histochemical Analyses

Following the treadmill exercise regimen, quadriceps muscle from EX and SED VCP^R155H/+^ and WT mice were harvested and embedded in cryo-sectioning mounting media (Electron Microscopy Sciences, Hatfield, PA) and placed into isopentane bath pre-chilled in liquid nitrogen. Samples were stored at −80°C before sectioning at 5–10 µm thickness. For immunohistochemical analyses, sections were stained with TDP-43, Ubiquitin, VCP, LC3-I/II, P62/*SQSTM1*, and COXIV specific antibodies (Abcam, Cambridge, MA). Subsequently, sections were washed with PBS and incubated with fluorescein-conjugated secondary antibodies (Sigma-Aldrich, St. Louis, MO) for 1 hour at room temperature and mounted with DAPI-containing mounting media (Vector Laboratories, Inc., Burlingame, CA) and analyzed by fluorescence microscopy. Additionally, Hematoxylin and Eosin (H&E), SDH and NADH staining was performed using routine methods and quantified by light microscopy (Carl Zeiss, Thornwood, NY) as described previously [35].

### Quantification of Protein Expression

Quadriceps muscle samples from exercise and sedentary VCP^R155H/+^ and WT mice were harvested and extracted using the NE-PER Nuclear and Cytoplasmic Extraction Kit (Thermo Scientific, Rockford, IL). Protein concentrations were determined using the Nanodrop according to the manufacturer's protocols. Equal amounts of proteins were separated on Bis-Tris 4–12% NuPAGE gels (Invitrogen Life Technologies Inc., Carlsbad, CA), and run on a mini Novex cell for 35 minutes at 200 V. The expression levels of proteins were analyzed by Western blotting using VCP, TDP-43, COXIV, P62/*SQSTM1*, LC3-I/II, and Ubiquitin-specific antibodies. Membranes were washed in TBST (0.5%) and probed with anti-mouse or anti-rabbit secondary antibodies for 1 hour at room temperature. Membranes were then washed and bands were detected using the Immun-Star™ WesternC™ Chemiluminescence Kit (Bio-Rad Laboratories, Hercules, CA) as described previously [35], [36]. Equal protein loading was confirmed by alpha tubulin antibody (Santa Cruz Biotechnology, Santa Cruz, CA) staining.

### TUNEL Analysis

Apoptosis in mouse quadriceps muscle tissue samples was analyzed by the DeadEnd Fluorometric TUNEL System (Promega, Madison, WI). For TUNEL analysis, muscle cryosections from EX or SED VCP^R155H/+^ mice and WT littermates were stained as described previously [34]. Briefly, cells were fixed in 4% paraformaldehyde for 15 minutes, washed in PBS for 5 minutes and permeabilized with 20 µg/ml Proteinase K solution for 10 minutes at room temperature. Cells were then washed in PBS for 5 minutes and 100 µl of equilibration buffer was added for 10 minutes. The cells were labeled with 50 µl of TdT reaction mix and incubated for 60 minutes at 37°C in a humidified chamber. Stop reaction was added for 15 minutes after which the cells were washed, counterstained, and prepared for analysis. The percentage of TUNEL+ cells was calculated.

### Statistics

All data are expressed as mean value ± standard error of the mean. Differences in continuous variables between exercised and sedentary WT and heterozygote VCP^R155H/+^ using one-way analysis of variance with pairwise *t*-tests corrected for multiple comparisons were compared. For categorical data, we used a chi-square test with pairwise tests using the Fisher's exact test. A probability value of ≤0.05 was considered statistically significant.

## Results

### Effects of treadmill exercise on muscle strength and running performance

**Figure 1:**
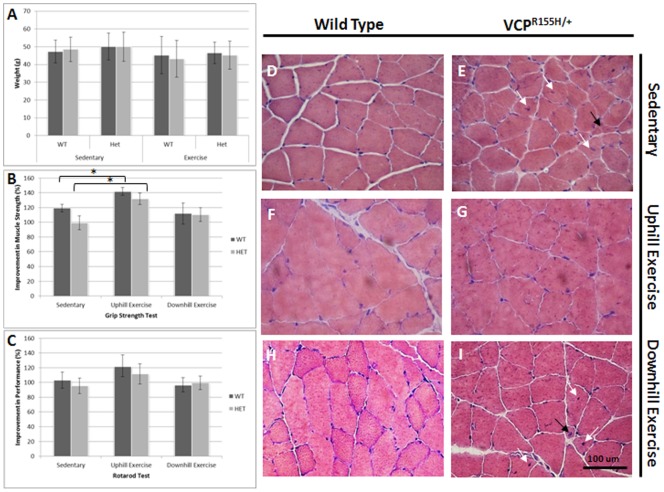
Weights, muscle strength measurements and histological analyses of sedentary and exercised VCP^R155H/+^ and WT mice. (A) Weight measurements of WT and VCP^R155H/+^ mice. (B) Muscle strength impairment observed by grip strength meter in SED VCP^R155H/+^ knock-in as compared with uphill exercised littermates and WT mice. (C) Physical performance as demonstrated a decline by Rotarod analysis in the sedentary VCP^R155H/+^ mice as compared with uphill exercised littermates and WT. Animal ages (months) (X axis) and relative values versus wild type littermates (Y axis). Statistically significant *p<0.05, **p<0.005, and ***p<0.001 values denoted. (D) H&E staining of quadriceps muscle from 19-month-old sedentary WT (nuclei depicted by arrows) and (E) sedentary *VCP* ^R155H/+^ knock-in mice. There was a significant decrease in muscle mass with wide variation in muscle fiber size, central nuclei, split fibers and extensive fiber necrosis (arrows) observed in sedentary mice as compared to uphill exercised animals. (F) H&E staining of quadriceps muscle from 19-month-old uphill exercised and (G) VCP^R155H/+^ animals. (H) H&E staining of quadriceps muscle from 19-month-old downhill exercised WT and (I) VCP^R155H/+^ mice (Magnification: 630X). The number of animals used was n = 8–10/group.

VCP-associated disease patients have slow progressive onset of myopathy involving the shoulder girdle muscles and hips. We characterized our transgenic 19-month old VCP^R155H/+^ animals and found features and characteristics typical of human IBMPFD disease [35]. The weight measurements of the VCP^R155H/+^ and WT before and after exercise were similar (**Figure 1A**). The running speed was increased as the mice became more trained over the course of the 2 month uphill and downhill exercise trainings for the VCP^R155H/+^ and WT littermates. The uphill exercised VCP^R155H/+^ and WT mice depicted an overall improvement in muscle strength and motor coordination measured by the grip strength and Rotarod tests compared to their sedentary counterparts, unlike the downhill exercised VCP^R155H/+^ and WT mice. The grip strength measurements of the uphill exercised VCP^R155H/+^ animals averaged a significantly elevated improvement of 32% relative to sedentary VCP^R155H/+^ animals and WT averaged an increased improvement of 22%. After the downhill exercise regimen, the WT mice depicted a slightly weaker muscle strength with a −7% decrease while the VCP^R155H/+^ mice demonstrated a slight improvement in muscle strength, however, neither were significantly different from the sedentary mice (**Figure 1B**). The Rotarod performance test revealed an increased average improvement of 16% in the VCP^R155H/+^ mice and 19% in WT mice for the uphill exercised animals analyzed. After the downhill exercise, the WT animals presented a weaker performance averaging a −6% decrease and 4% improvement in the VCP^R155H/+^ with the Rotarod performance, albeit, neither of which were significantly different from the sedentary mice (**Figure 1C**).

### Muscle phenotype in VCP^R155H/+^ after exercise

Since myofiber atrophy is a major cause of muscle weakness, we examined whether fiber size change occurred as a result of the exercise regimen. Quadriceps muscles were harvested from the exercise and sedentary WT and VCP^R155H/+^ mice (**Figure 1D,E**) for histological analysis. Remarkably, the uphill exercised VCP^R155H/+^ animals demonstrated significant improvements in their muscle architecture including quadriceps fiber size, decreased endomysial space, and decreased centrally localized nuclei (**Figure 1F,G** **and** **Table 1**) as compared to VCP^R155H/+^ sedentary mice, which display angular and atrophic fibers, endomysial fibrosis and centralized nuclei (**Figure 1E**) (shown by arrows). However, the downhill exercised mice did not demonstrate any significant improvement in the muscle quadriceps architecture (**Figure 1H,I**) (shown by arrows).

**Table 1:** Percentage of fibers with centralized nuclei and inclusion bodies over total fibers in 19-month old sedentary versus uphill exercised VCP^R155H/+^ mice.

Experiment Group	Histological Methodology	Observed Organelle	Wild Type (%)	VCP^R155H/+^ (%)	Cells Counted (WT/VCP^R155H/+^)
*Sedentary*	Hematoxylin and Eosin	Centrally located nuclei	2.27±1.00	6.13±0.90	1500/1500
	TDP-43/Ubiquitin Immunohistochemistry	Inclusion bodies	3.23±0.81	15.71±1.01	800/750
*Uphill Exercise*	Hematoxylin and Eosin	Centrally located nuclei	2.33±0.70	6.07±0.30	1500/1500
	TDP-43/Ubiquitin Immunohistochemistry	Inclusion bodies	2.29±0.44	11.59±1.91	750/750

Significant differences are denoted by p<0.05.

### Effects of exercise training on autophagy cascade

**Figure 2:**
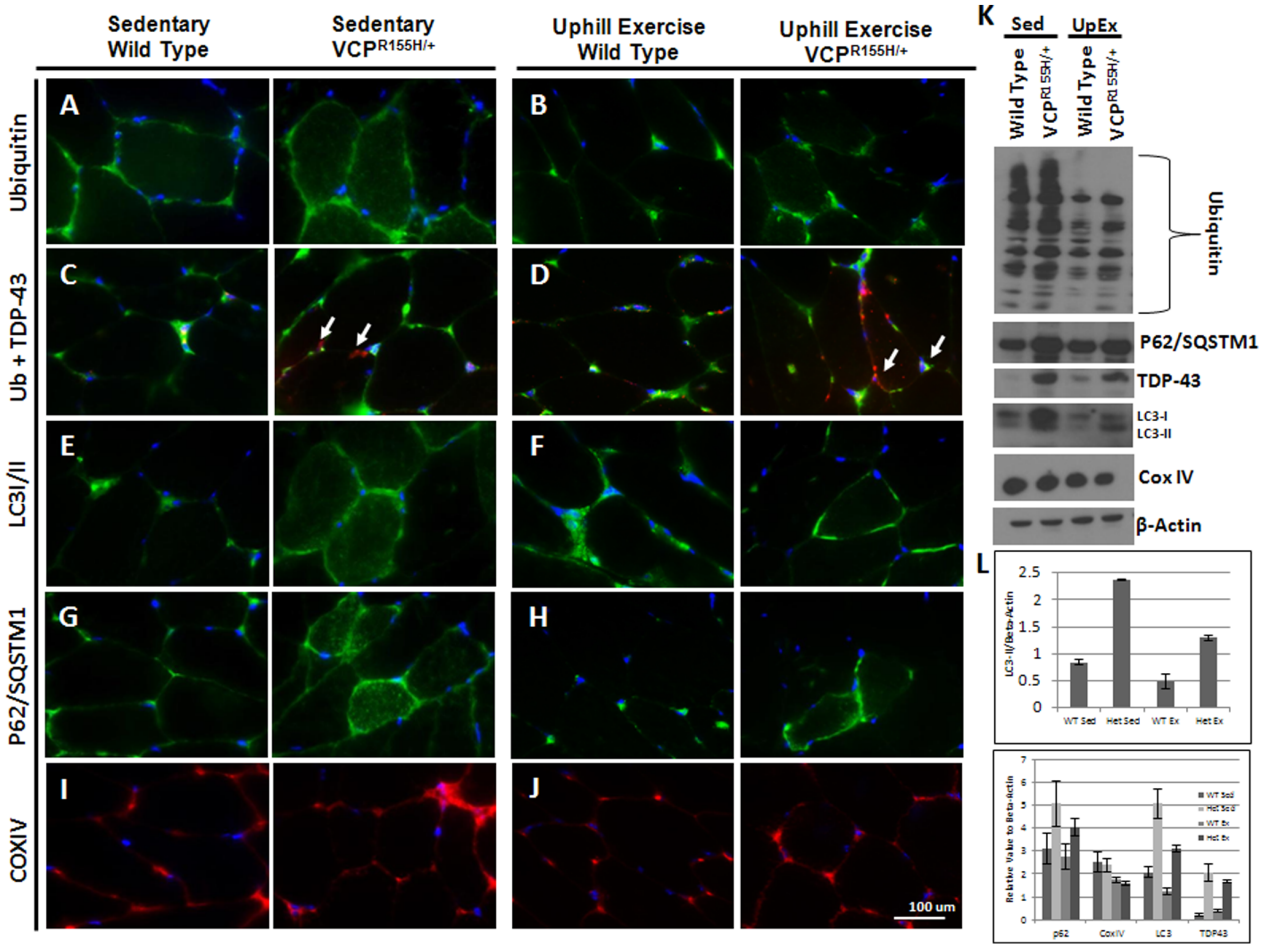
Immunohistochemical analyses of autophagy cascade in sedentary and exercised VCP^R155H/+^ mice. Immunohistochemical analysis of sedentary and exercised WT and VCP^R155H/+^ mice stained with (A,B) Ubiquitin, (C,D) P62/SQSTM1, (E,F) COXIV, (G,H) LC3-I/II, (I,J) Ubiquitin and TDP-43 (arrows pointing to TDP-43 localization) (Magnification: 630X). (K) Western blot and (L) densitometry analyses of these proteins demonstrated lower levels of LC3-I/II, Ubiquitin, P62/*SQSTM1*, and TDP-43 upon the uphill exercise regimen in VCP^R155H/+^ animals (Magnification: 630X). Statistical significance is denoted by *p<0.05, **p<0.005, and ***p<0.001. The number of animals used was n = 8–10/group.

Autophagy is highly essential for the maintenance of cellular homeostasis and for the removal of intracellular waste products. This process has been found to be impaired in several degenerative diseases including myopathies. Corroborating our previous findings [34], [35], quadriceps of sedentary VCP^R155H/+^ mice showed higher expression levels of ubiquitin, P62/*SQSTM1*, LC3I/II, and TDP-43 (**Figure 2A,C,E,G**), while uphill exercised VCP^R155H/+^ animals demonstrated lower levels of these autophagy proteins, indicating an improvement, compared to the sedentary littermates (**Figure 2B,D,F,H**). COXIV expression levels did not differ between sedentary WT and VCP^R155H/+^ mice, but mice on the uphill exercise regimen showed a modest reduction in COXIV levels (**Figure 1I,J**). Western blot analyses of these autophagy proteins in WT and VCP^R155H/+^ animals confirmed these findings (**Figure 2K**). Interestingly, there was a substantial decrease in the overall LC3 expression in the uphill exercised VCP^R155H/+^ mice, but more importantly there was a striking decrease in LC3-II expression (**Figure 2K,L**). Additionally, after quantification of ubiquitin and TDP-43 positive inclusion bodies, VCP^R155H/+^ mice presented a decrease in the percentage of muscle fibers expressing these pathological inclusion bodies (Table 1). No changes in VCP expression were found in exercised VCP^R155H/+^ animals (data not shown).

### Mitochondrial enzyme complexes in VCP^R155H/+^ mice after treadmill exercise

**Figure 3:**
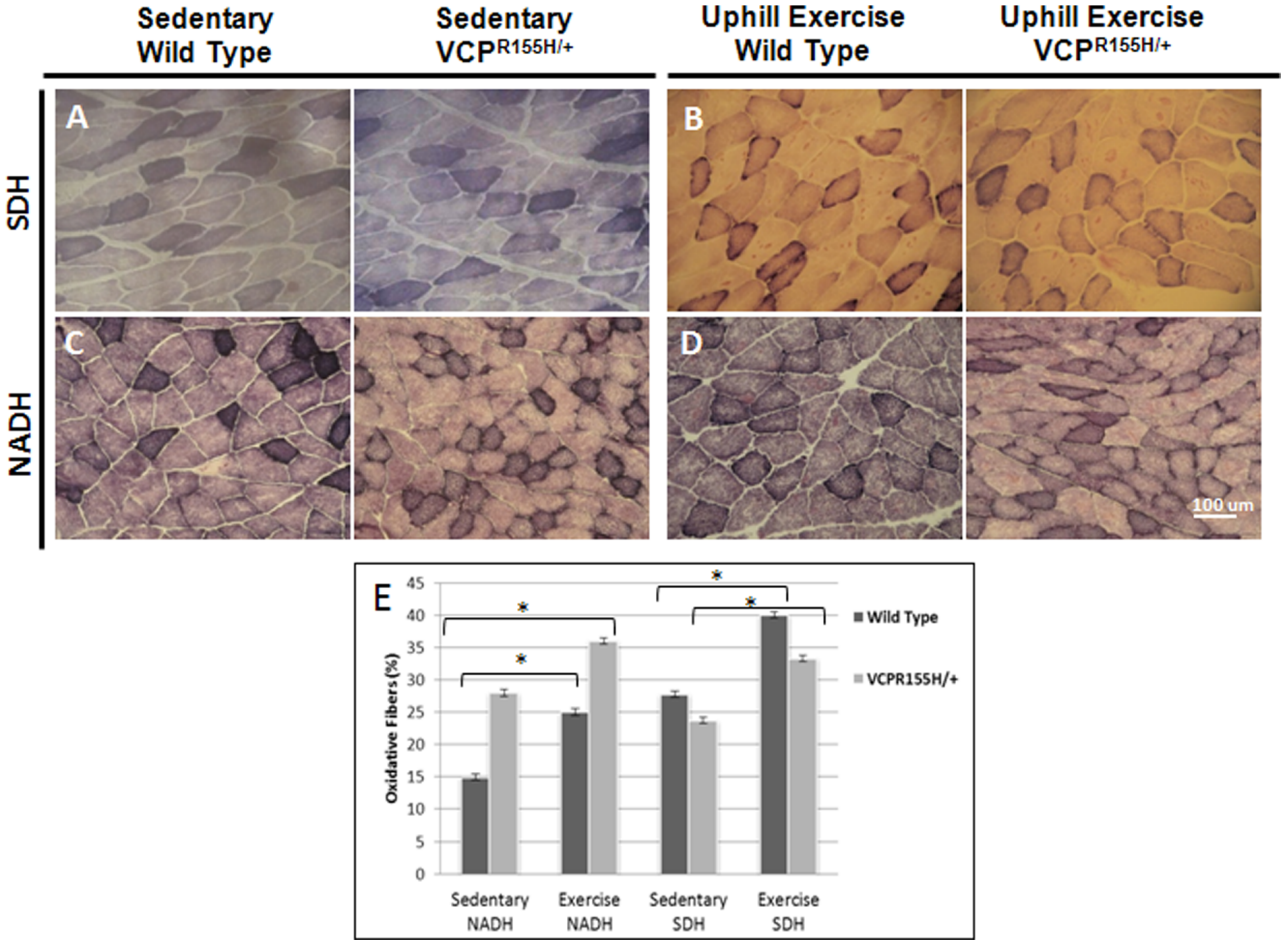
Mitochondrial enzyme analysis of sedentary and exercised VCP^R155H/+^ mice. Quadriceps muscles from WT and VCP^R155H/+^ mice at 19-months of age (A) sedentary and (B) exercised were stained with SDH. Quadriceps muscles from WT and VCP^R155H/+^ mice (C) sedentary and (D) exercised stained with NADH antibody. (E) Quantification of SDH and NADH type I and II quadriceps fibers in sedentary and exercised WT and VCP^R155H/+^ mice (Magnification: 630X). The number of animals used was n = 8–10/group.

Identification of oxidative and non-oxidative fibers by SDH and NADH staining was used in assessing mitochondrial pathology, whereby increased oxidative fibers may be a hallmark of centronuclear myopathy in mice and humans. However, studies on exercise training in horses demonstrated increased oxidative capacity and attenuation of exercise-induced ultrastructural damage in skeletal muscles [37]. Therefore, we examined the effects of uphill exercise training on the mitochondrial complexes of VCP^R155H/+^ and WT animals quadriceps muscles. Quadriceps from exercised VCP animals stained with succinic dehydrogenase (SDH) depicted “purple-speckled” fibers when compared to WT littermates (**Figure 3A,B**). Compared to WT littermates which depicted a normal mosaic staining pattern, nicotinamide adenine dinucleotide (NADH) staining of sedentary heterozygous VCP^R155H/+^ mice quadriceps revealed decreased staining (lighter fibers) (**Figure 3C,D**). Interestingly, after exercise, both WT and VCP^R155H/+^ animals revealed comparable numbers of SDH and NADH oxidative fibers, suggestive of increased oxidative potential (**Figure 3E**).

### Effect of treadmill exercise on apoptosis

**Figure 4:**
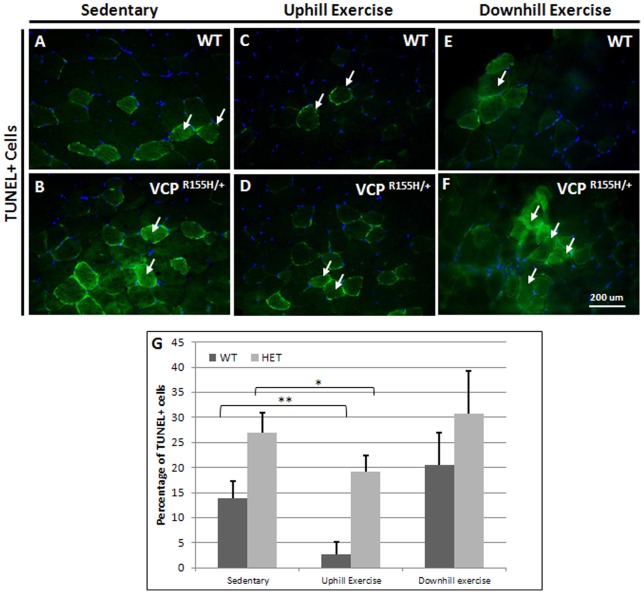
TUNEL analyses of quadriceps in sedentary and exercised VCP^R155H/+^ mice. TUNEL staining of quadriceps muscles from sedentary (A) WT and (B) VCP^R155H/+^; exercised (C) WT and (D) VCP^R155H/+^ mice at 19-months of age. Quadriceps muscles from (E) WT and (F) downhill exercised VCP^R155H/+^ mice were stained with TUNEL (Magnification: 630X). (G) Quantification of TUNEL in the sedentary and exercised mice. Statistical significance is denoted by *p<0.05, **p<0.005, and ***p<0.001 by Student one-tailed t test. The number of animals used was n = 8–10/group.

To examine the effect of exercise on apoptosis, terminal deoxynucleotidyl transferase dUTP nick end labeling (TUNEL) staining was performed on the quadriceps sections of WT and VCP^R155H/+^ mice. Through the uphill exercise regimen, as compared to sedentary control littermates (**Figure 4A,B**), muscle fibers of the VCP^R155H/+^ mice displayed reduced levels of apoptosis, as there were significantly fewer TUNEL positive cells in uphill exercised WT and VCP^R155H/+^ quadriceps (**Figure 4C,D**). The number of TUNEL-positive cells in the quadriceps was 14% in the WT sedentary group, 27% in the VCP^R155H/+^ sedentary group, 3% in the WT uphill exercised group, and 19% in the VCP^R155H/+^ uphill exercised group. In the downhill exercised groups, the number of TUNEL+ cells remained the same (**Figure 4E,F**). Quantification of TUNEL staining confirmed these results, indicating uphill treadmill exercise exerted a statistically significant effect on DNA fragmentation in the quadriceps muscles of VCP^R155H/+^ mice (**Figure 4G**).

## Discussion

Despite significant progress, there are no treatment modalities available for patients dying from VCP-associated myopathy. The cellular and molecular mechanisms underlying these neurodegenerative diseases remain unknown. Our recent microarray studies in muscle biopsies indicate that multiple signaling pathways are dysregulated in patients with VCP-associated inclusion body myopathy (IBM), which include abnormalities in actin cytoskeleton, ErbB signaling, cancer, regulation of autophagy and lysosomal signaling transduction cascades [38]. Previous studies in patient myoblasts have implicated impaired autophagy in the pathogenesis of VCP disease [39], [40].

Several reports point out the beneficial effects of exercise training in muscular dystrophies since it plays a critical role in many processes including intracellular calcium, activation of signaling pathways, and increased antioxidant capacity. However, the molecular mechanisms by which exercise may delay or reverse skeletal muscle myopathy remains elusive. Skeletal muscle is a highly adaptable tissue and changes in response to environmental stimuli [41]. From a pre-clinical perspective, this type of physical exercise activity is not harmful and may also provide possible mechanisms as to the benefits of endurance training. Through the findings of this exercise protocol, autophagy is functional in the VCP^R155H/+^ mice running uphill as shown with the decrease in the expressions of protein autophagy markers (ubiquitin, P62/*SQSTM1*, and LC3I/II) that typically accumulate in the VCP mutant mice. The decrease in LC3-II levels in the exercise group further demonstrates that there may be an increase in autophagy since LC3-II itself is degraded through autophagy after being converted from LC3-I. There was also a decrease in the ubiquitin and TDP-43 positive inclusion bodies in the uphill exercised VCP^R155H/+^ mice indicative of the pathological cellular debris being broken down. In response to cellular stresses such depletion of cellular ATP, the upregulated AMPK pathway promotes further autophagic proteolysis through inhibition of the mTOR pathway [42]. With the AMPK pathway being upregulated in exercise, cellular homeostasis may become more established by recycling excess cellular debris that is cytotoxic. In the uphill exercised VCP^R155H/+^ mice, exercise-induced increase of autophagic proteolysis may have resulted in a reduction of cell death in muscle fibers.

A wealth of information points to training exercise platforms as a key intervention for prevention and treatment in neurodegenerative diseases in patients. In Becker muscular dystrophy (BMD), endurance training has depicted improvement in fitness and strength by 13–40% in patients [43]. A further study by this group [44] examined resistance training in patients with limb girdle and BMDs and found that exercise increased the muscle strength and endurance in some of the muscles in patients. These two studies concluded that endurance training is a safe method to increase exercise performance in patients with BMD and may serve as an approach to rehabilitation. Another study found that progressive resistance exercise improved physical and strength performance and may serve as an effective and worthwhile treatment in people with mild and moderate Parkinson's disease [45]. Siciliano et al. (2012) demonstrated the positive effects of aerobic training in muscle performance in patients with mitochondrial myopathies [46].

This report investigates the effects and consequences of both uphill and downhill exercise physiology in the VCP^R155H/+^ heterozygote and WT mouse model compared to age-matched and sex-matched sedentary control littermates by Rotarod performance, grip strength measurements and by histological analyses. Findings from this study indicate that a 6-week study low-intensity uphill treadmill exercise regimen can improve the whole-body strength, and mitochondrial oxidative capacity of the quadriceps muscles of the VCP^R155H/+^ heterozygote mice. There was no indication that muscle was detrimentally affected by uphill exercise. In contrast, the downhill exercised mice demonstrated no significant improvement in muscle strength, performance and pathology. Our findings indicated that only an uphill treadmill endurance training improved the muscle strength, pathology and biochemical markers for autophagy and apoptosis observed in the heterozygote VCP^R155H/+^ disease model.

## Conclusions

Thus, this investigation explored the effects of exercise as a potential therapy to help slow the progression of neurodegenerative diseases, such as IBMPFD and ALS. Endurance training did not depict any impairment or injury to the muscle in the VCP^R155H/+^ mouse model. In conclusion, future studies will explore exercise training as a potential platform for therapeutic purposes and/or advancements in patients with VCP-associated diseases in hopes of reversing the pathogenetic mechanism(s) underlying these neurodegenerative disorders.
